# Distinct epigenetic signatures of classical and hypervirulent *Klebsiella pneumoniae*

**DOI:** 10.1128/msphere.00464-23

**Published:** 2023-12-19

**Authors:** Dipannita Ghosh, Arijit Pal, Sarita Mohapatra, Stephen Raj, Perumal Vivekanandan

**Affiliations:** 1 Kusuma School of Biological Sciences, Indian Institute of Technology Delhi, New Delhi, India; 2 Department of Microbiology, All India Institute of Medical Sciences, New Delhi, India; 3 Department of Microbiology, PGIMER, Chandigarh, India; University of Kentucky College of Medicine, Lexington, Kentucky, USA

**Keywords:** hypervirulent *Klebsiella pneumoniae*, bacterial epigenetics, methylation, virulence

## Abstract

Emergence and spread of the hypervirulent pathotype of *Klebsiella pneumoniae* have significantly increased infection rates in community as well as healthcare settings. There is an increasing interest to identify discriminating features between classical *K. pneumoniae* (cKp) and hypervirulent *K. pneumoniae* (hvKp) to facilitate our understanding of the rapid emergence and dissemination of the hypervirulent pathotype. Here, we sought to identify unique epigenetic signatures of hvKp pathotype that differ from its classical counterpart using single-base resolution methylome analysis of native DNA sequencing on the Oxford Nanopore Technologies platform. The overall global adenine methylation in GATC motifs (i.e., Dam methylation motif) and cytosine methylation in CCWGG motifs (i.e., Dcm methylation motif) were significantly higher in hvKp isolates compared to that in cKp isolates, irrespective of their position in chromosomes or putative extra-chromosomal genetic elements. Notably, we observed significant enrichment of hypermethylated GATC and CCWGG motifs in the virulome of hvKp compared to hvKp genes not directly associated with virulence. We also observed increased methylation of GATC and CCWGG motifs in the capsule synthesis locus of hvKp isolates compared to cKp isolates. Furthermore, we identified several differentially methylated genes (DMGs) between the two pathotypes; interestingly, these DMGs include metal ion transporters, multidrug efflux pumps, transcriptional regulators of stress response, and genes associated with biofilm formation. Our results highlight hypermethylation of GATC and CCWGG motifs as unique epigenetic signatures of hvKp isolates.

**IMPORTANCE:**

Hypervirulent *Klebsiella pneumoniae* (hvKp) is a more virulent and rapidly evolving hypermucoviscous pathotype of classical *K. pneumoniae* (cKp). The hypervirulent pathotype is a major public health concern and is associated with high infection rates in community as well as hospital settings. With the recent emergence of multidrug-resistant hvKp, it has become imperative to investigate non-classical mechanisms such as epigenetics in addition to canonical biochemical and genetic mechanisms that delineate and differentiate the hypervirulent pathotype from its classical counterpart. Here, we identify genome-wide differences in adenine and cytosine methylation marks at well-characterized motifs between the two pathotypes. Overall, significantly higher levels of methylation were observed across chromosomal DNA and extrachromosomal elements in hvKp compared to cKp. Among hvKp isolates, the genes associated with virulence are particularly enriched for methylation marks. Our findings shed light on how epigenetic signatures may help distinguish the pathogenic potential of bacteria.

## OBSERVATION

Epigenetic inheritance is known to influence several phenomena in bacterial survival and pathogenicity (1). Methylation of adenine [i.e., N6-methyladenine (m^6^A)] and cytosine [i.e., 5-methylcytosine (m^5^C)] is not only involved in regulation of gene expression but is also known to affect mutational rates and processes associated with antimicrobial drug resistance and virulence in bacteria (1). There are two pathotypes of *Klebsiella pneumoniae*: classical (cKp) and hypervirulent (hvKp) (2). HvKp has been associated with highly invasive infections affecting multiple organs and is rapidly spreading worldwide. Here, we investigate the differences in the epigenetic signatures between hvKp and cKp. Genomic DNA isolated from five cKp and six hvKp clinical isolates (3) was used to perform native DNA whole-genome sequencing (WGS) using Oxford Nanopore MinION. The six hvKp isolates, identified using string test and PCR for *iucA* gene, were previously characterized for antimicrobial resistance genes and hypervirulence genes by WGS (3). Assessment of growth under laboratory conditions showed no significant difference between the cKp and hvKp isolates studied here (Fig. S1). Polished *de novo* assemblies for each of the 11 isolates obtained after nanopore sequencing were assessed for N50, depth, and completeness (Table S1) and subsequently used to perform modified basecalling to identify m^6^A and m^5^C methylation frequencies at GATC (methylated by DNA adenine methylase or Dam) and CCWGG (methylated by DNA cytosine methylase or Dcm) in the genomes of the cKp and hvKp isolates.

Both GATC motif and CCWGG motif frequencies were significantly higher in chromosomal contigs (contigs > 5 Mb) compared to those in putative extra-chromosomal genetic elements (PEGEs; contigs < 5 Mb) for both cKp and hvKp isolates sequenced (Fig. S2A and B). Higher GATC frequencies have been reported in chromosomal DNA compared to PEGEs (4). Depletion of GATC as well as CCWGG motif frequencies in the extra-chromosomal elements, observed in both cKp and hvKp, is consistent with the premise that these motifs are not involved in host defense in *K. pneumoniae*.

**Fig 1:**
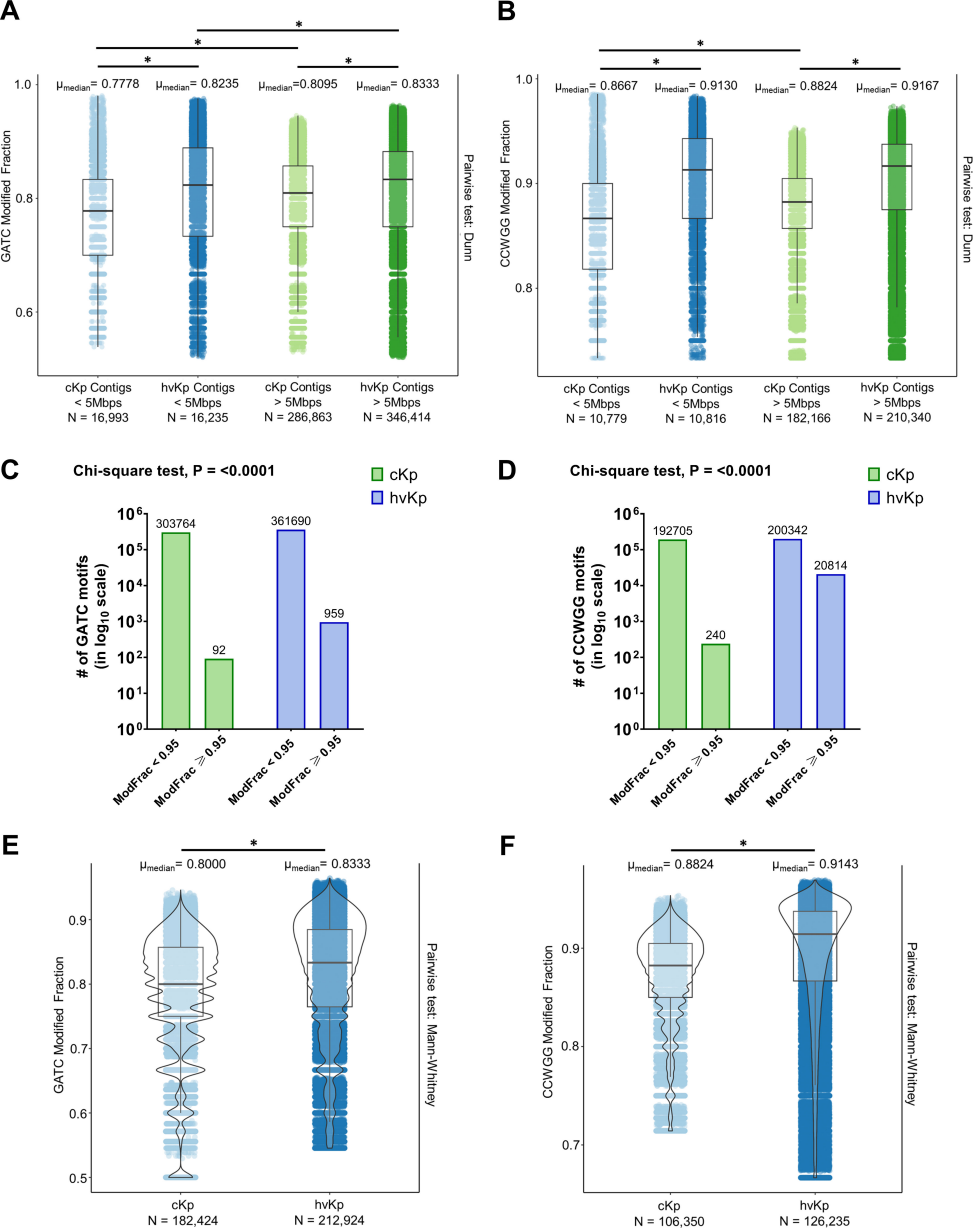
hvKp has higher levels of methylation in GATC and CCWGG motifs compared to cKp. (**A** and **B**) Comparison of the overall GATC (**A**) and CCWGG (**B**) ModFrac values between the chromosomes (contigs > 5 Mb) and PEGEs (contigs < 5 Mb) of cKp and hvKp, showing higher overall methylation of both cytosines and adenines in hvKp compared to cKp. (Outliers, defined using 1.5 times interquartile range method, have been removed.) *P < 0.0001. (**C** and **D**) Bar graphs depicting enrichment of hypermethylated GATC (**C**) and CCWGG (**D**) motifs (i.e., motifs with ModFrac values ≥ 0.95) in hvKp compared to cKp. (**E** and **F**) Violin plots depicting the ModFrac values of GATC (**E**) and CCWGG (**F**) motifs present within the common core genes of the cKp and hvKp isolates. (Outliers, defined using 1.5 times interquartile range method, have been removed.) *P < 0.0001.

We analyzed the modified fraction (ModFrac) values of GATC and CCWGG motifs across all contigs in cKp and hvKp (Fig. 1A and B). The overall methylation level in hvKp isolates is significantly higher compared to that in cKp for both chromosomal contigs and PEGEs. Within hvKp, the GATC ModFrac values are modestly high in chromosomal contigs compared to PEGEs (medians 0.8333 vs 0.8235; *P* < 0.0001). The same trend is also observed for cKp (medians 0.8095 vs 0.7778; *P* < 0.0001). Also, we found that the CCWGG methylation levels in hvKp are significantly higher compared to cKp for both chromosomal (>5 Mb) and PEGE (<5 Mb) contigs. Interestingly, in hvKp isolates, hypermethylated GATC motifs (i.e., ModFrac values ≥ 0.95) were enriched by over 10-fold, and hypermethylated CCWGG motifs (i.e., ModFrac values ≥ 0.95) were enriched by over 86-fold as compared to that in cKp isolates (Fig. 1C and D). This finding indicates that hypermethylation of GATC and CCWGG motifs is a unique epigenetic signature of hvKp isolates. Furthermore, the analysis of the core genome (common to both pathotypes) indicates higher global methylation levels of both adenine and cytosine methylation levels in hvKp isolates compared to cKp isolates (Fig. 1E and F). Therefore, increased methylation of GATC and CCWGG motifs in hvKp chromosomal contigs is independent of the accessory genome and the differences in motif frequency or location.

Next, we compared the distribution of ModFrac values of GATC and CCWGG motifs across different genetic features of the cKp and hvKp assemblies. Analysis of methylation profiles of 200-bp upstream region of gene-coding sequences showed higher methylation levels for both GATC and CCWGG motifs in hvKp (Fig. S3). Significantly higher levels of GATC methylation were found in the gene bodies than in intergenic regions for chromosomal contigs and PEGEs among hvKp isolates but not cKp isolates (Fig. S4A). While higher levels of CCWGG methylation were found in the intergenic regions compared to gene bodies for chromosomal contigs of hvKp, the same was not true for cKp (Fig. S4B). In addition, cytosine methylation was more pronounced in gene bodies than in the intergenic regions of PEGEs in both hvKp and cKp isolates. These findings highlight the differences between adenine and cytosine methylation levels at specific genomic regions and PEGEs in hvKp isolates.

A hypermucoviscous colony phenotype and increased capsule production have been linked to hvKp (5). As the capsule synthesis (cps) locus harbors genes essential for capsular polysaccharide synthesis in *K. pneumoniae*, we assessed the methylation profile of genes present within this locus in our cKp and hvKp isolates. For GATC motifs, significantly higher methylation levels were seen selectively in the coding strand of the cps locus genes of hvKp isolates compared to that in cKp (Fig. S5A). In the case of CCWGG motifs, both the template and coding strands of cps locus genes had higher cytosine methylation in hvKp than cKp (Fig. S5B). Since Type 1 and Type 3 fimbriae are important for virulence in *Klebsiella*, we also assessed the methylation levels of the conserved fimbriae-encoding cluster (6) (Fig. S6). The hvKp isolates have higher methylation levels for both GATC and CCWGG motifs across the fimbriae-encoding cluster compared to cKp.

**Fig 2:**
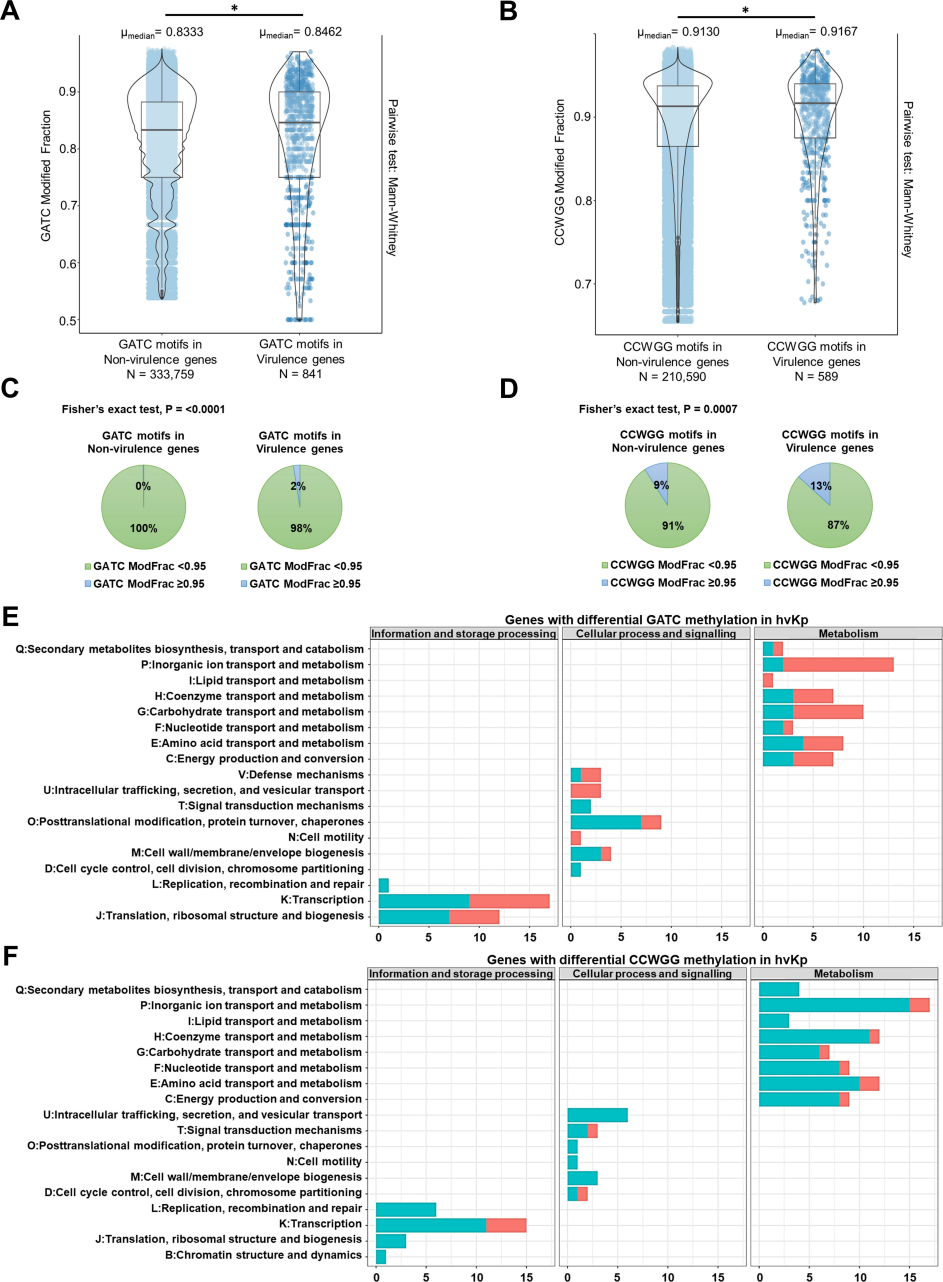
Methylation profile of virulence genes and identification of DMGs in hvKp. Violin plots of ModFrac values of GATC (**A**) and CCWGG (**B**) mapping to virulence and non-virulence genes of the six hvKp isolates. (Outliers, defined using 1.5 times interquartile range method, have been removed.) *P < 0.01. (**C** and **D**) Pie charts depicting the proportion of hypermethylated GATC (**C**) and CCWGG (**D**) motifs (i.e., motifs with ModFrac values ≥ 0.95) being significantly higher in the virulence genes compared to genes not directly associated with virulence in hvKp. (**E** and **F**) Genes differentially methylated for GATC (**E**) and CCWGG (**F**) motifs in hvKp (compared to cKp) were classified into COGs of proteins. Hypomethylated genes are depicted in cyan, while hypermethylated genes are represented in red. Genes with unknown functions were excluded from the plots.

Hypervirulent *K. pneumoniae* harbors genetic markers that are known to contribute to increased virulence, such as *iucA*, *iroB*, *rmpA*, *rmpA2*, and *peg-344* (2). The ModFrac values for both adenine methylation in GATC (Fig. 2A) and cytosine methylation in CCWGG (Fig. 2B) motifs are significantly higher for the virulome (acquired virulence genes) compared to those in the rest of the hvKp genes (*P* < 0.01). In addition, the proportion of hypermethylated (ModFrac ≥ 0.95) GATC and CCWGG motifs was significantly higher in the virulome compared to the rest of the genes in hvKp isolates (*P* < 0.001; Fig. 2C and D). The virulome of the hvKp isolates sequenced in this study includes genes encoding salmochelin (*iroBCDN*), aerobactin (*iucABC* and *iutA*), yersiniabactin (*ybtPQ*), microcin (*mchF*), and capsule activator (*rmpA2*). The increased methylation of the hvKp virulome merits further investigation.

Finally, we investigated the differentially methylated genes (DMGs) by calculating the *z*-scores of the difference in ModFrac values of cKp and hvKp for each core gene (Fig. S7). For adenine GATC methylation, we found 71 hypermethylated (z-score ≥ 2) and 73 hypomethylated (z-score ≤ 2) genes in hvKp. For cytosine CCWGG methylation, we found only 17 hypermethylated core genes, while the number of hypomethylated genes was as high as 114. Furthermore, functional analysis of the differentially methylated core genes using Clusters of Orthologous Groups (COGs) of protein classification revealed that hvKp DMGs for both GATC and CCWGG motifs were predominantly associated with metabolism (GATC: 51 genes; CCWGG: 73 genes), followed by information and storage processing (GATC: 30 genes; CCWGG: 25 genes) (Fig. 2E and F). Inorganic ion transport, especially metal ions such as iron, manganese, zinc, and copper, is crucial for bacterial virulence (7). Interestingly, genes involved in inorganic ion transport and metabolism showed reciprocal methylation profiles in hvKp for adenines and cytosines; there were 11 hypermethylated and 2 hypomethylated genes for adenine GATC methylation, whereas for methylation of cytosines in CCWGG motifs, we observed 2 hypermethylated and 15 hypomethylated genes. Among the GATC-DMGs belonging to the inorganic ion transport and metabolism COG category (Table S2A), we found genes associated with metal ion transport, including copper (*cutF*), zinc (*znuA*), and manganese (*mntP* and *mntC*). Some of the GATC-DMGs in this category also belonged to major facilitator superfamily transporters, including *emrD* gene, which encodes a multidrug efflux protein associated with antimicrobial resistance and biofilm formation (8), and *yhjX*, transporter known to mediate adaptive resistance to gentamicin (9). Among CCWGG-DMGs in this COG category (Table S2B), we found genes encoding proteins involved in the transport of iron (*sitC* and *fhuB*) and zinc (*znuA* and *znuB*) along with a cation exchanger (*yrbG*) linked with capsular lipopolysaccharide biogenesis (10).

We also found several hvKp DMGs involved in transcription. Notable hvKp genes differentially methylated for adenine GATC include *feoC* [upregulates an Fe(II) transporter (11)], *marA* [upregulates multidrug efflux (12)], *rfaH* [transcription antitermination protein associated with virulence (13)], *matA* [fimbriae activator; promotes adhesion and represses motility (14)], *pspC* [phage shock protein C associated with virulence (15)], *mprA* [negative regulator of multidrug efflux pumps (16)], *yieP* [regulates tolerance to organic acids (17)], and *leuO* [global transcription factor associated with biofilm formation and virulence (18)] (Table S3A). In the case of CCWGG motif methylation, noteworthy DMGs in hvKp include *marA*, *yieP*, *ywbI* [transcriptional regulator involved in biofilm formation (19)], *yebC* [associated with regulation of surface polysaccharides and virulence (20)], and *ramA* [regulates stress response implicated in multidrug resistance (12)] (Table S3B).

Our findings show that in this cohort of *K. pneumoniae* clinical isolates, hvKp has overall higher levels of adenine (GATC) and cytosine (CCWGG) methylation compared to its classical counterpart. Furthermore, adenine and cytosine methylation levels in the cps locus are higher for hvKp isolates compared to cKp isolates. Of note, hypermethylation at the GATC and CCWGG motifs is more pronounced in the virulome of hvKp than in the rest of the hvKp genes. We also identified several DMGs in hvKp, including genes encoding metal ion transporters, multidrug efflux pumps and their regulators, surface polysaccharide regulators, and transcriptional regulators of stress response and biofilm formation. In sum, this work highlights the existence of previously unrecognized epigenetic differences between pathotypes of a given bacterial species.

## MATERIALS AND METHODS

### Genomic DNA isolation and quality control

Ethical permission from the institute (approval number IECPG-441/27.06.2019) and informed consent from all the patients who participated were obtained for conducting this study. Genomic DNA was extracted from overnight colonies of five cKp and six hvKp [previously characterized for antimicrobial resistance and virulence genes (3)] using QIAamp DNA Mini Kit (Qiagen). Supplier instructions were followed during the isolation process, which involved suspending the colonies in cell lysis buffer followed by Proteinase K and RNase A treatment, washing and elution of the DNA using spin columns. Qubit dsDNA BR Assay Kit (Thermo Fisher Scientific, USA) was used for quantification in the Qubit 2.0 Fluorometer. Horizontal agarose gel electrophoresis and ImplenNanoPhotometer N60 (Implen, Germany) were used to assess the DNA quality and integrity.

### Preparation of genomic libraries and sequencing

Native Barcoding Expansion Kit (EXP-NBD104) and Ligation Sequencing Kit (SQK-LSK109) from Oxford Nanopore Technologies (ONT), UK, were used to prepare native DNA libraries from the genomic DNA extracted. One microgram of DNA per sample was processed according to the manufacturer’s protocol. AmPure beads (Beckman Coulter, USA) were used for the washing steps in between the protocols. The libraries were loaded onto R9.4.1 flow cells (FLO-MIN106D) and sequenced on a MinION through the MinKNOW application.

### Basecalling, construction of *de novo* assemblies and quality control

The raw FAST5 files generated after sequencing were used for basecalling in Guppy (version 6.1.7) (ONT) with the high-accuracy model (dna_r9.4.1_450bps_hac.cfg), along with adapter trimming and demultiplexing the barcoded reads. Initial quality assessment of the FASTQ outputs was performed in Nanoplot (21). *De novo* assemblies were made from reads with Q score ≥10 for each of the 11 isolates using Flye v. 2.9.1 (22), followed by polishing of the consensus sequences in Medaka v1.7.1 by ONT. The completeness of the polished genome assemblies was assessed using BUSCO v.5.4.0 (23) with “enterobacterales_odb10” as the lineage data set.

### Annotation of *de novo* assemblies, identification of core genes and virulence genes

The polished assemblies were annotated in Prokka v1.14.5 (24), followed by pan-genome analysis using Roary (25) to identify the core genes present in all the five cKp and six hvKp assemblies. The assembled genomes were also used to find virulence and antimicrobial resistance genes using AMRFinderPlus (NCBI) (26).

### Methylation analysis

Prior to modified basecalling in Tombo v.1.5.1 (27) by ONT, ont_fast5_api (https://github.com/nanoporetech/ont_fast5_api) was used to convert the multiread FAST5 files to single-read files. The polished *de novo* assemblies were used as references to map the raw signals containing the basecalls to find positional information of the modified bases. “alternative_model” was used to perform GATC and CCWGG motif-specific modified base detection in the Tombo suite. The global ModFrac values of the GATC and CCWGG motifs were compared and visualized using the R packages ggplot2 (https://ggplot2.tidyverse.org/) and ggstatsplot (28). For core genome methylation analysis, we mapped the GATC and CCWGG motif positions identified by Tombo to the core genes before analyzing the distribution with ggstatsplot. Virulence gene methylation analysis was performed by mapping the GATC and CCWGG motif positions in the individual hvKp assemblies to the virulence genes identified by AMRFinderPlus and removing the same sites from the files where motif sites were mapped to all the genes of the hvKp assemblies. This was followed by distribution analysis using ggstatsplot.

### Identification of DMGs

The ModFrac values for both GATC and CCWGG motifs were averaged for all core genes of cKp and hvKp isolates identified previously. Only those genes were processed, which were present in more than one isolate of cKp and hvKp groups. The three-color heatmaps depicting the average GATC and CCWGG ModFrac values of each core gene were constructed in GraphPad Prism 9.0. Z-score analysis was performed using “scale ()” function of base R. The coding sequences (CDS) of DMGs identified using z-scores were used for COGs of protein classification in eggNOG-mapper v2 (29) with a minimum query coverage set to 80%. ggplot2 was used for visualization of the DMGs.

### Statistical analysis

All statistical analyses were carried out using ggstatsplot package in R and GraphPad Prism v.9. Results are presented within the figures with details in their respective legends.

## Data Availability

All native genomic DNA sequencing data have been made available from Sequence Read Archive (SRA), NCBI under BioProject ID PRJNA999042.

## SUPPLEMENTAL MATERIAL

Supplemental figures and tables msphere.00464-23-s0001.docx Figures S1 to S7 and Tables S1 to S3.

ASM does not own the copyrights to Supplemental Material that may be linked to, or accessed through, an article. The authors have granted ASM a non-exclusive, world-wide license to publish the Supplemental Material files. Please contact the corresponding author directly for reuse.
