# Supplementary material for: Distinct epigenetic signatures of classical and hypervirulent Klebsiella pneumoniae
Source: mSphere. 2023 Dec 19;9(1):e00464-23. doi: 10.1128/msphere.00464-23 (PMC10826340; doi:10.1128/msphere.00464-23)
Supplement: Supplemental figures and tables — Figures S1 to S7 and Tables S1 to S3. [file msphere.00464-23-s0001.docx]

**SUPPLEMENTARY FIGURES**


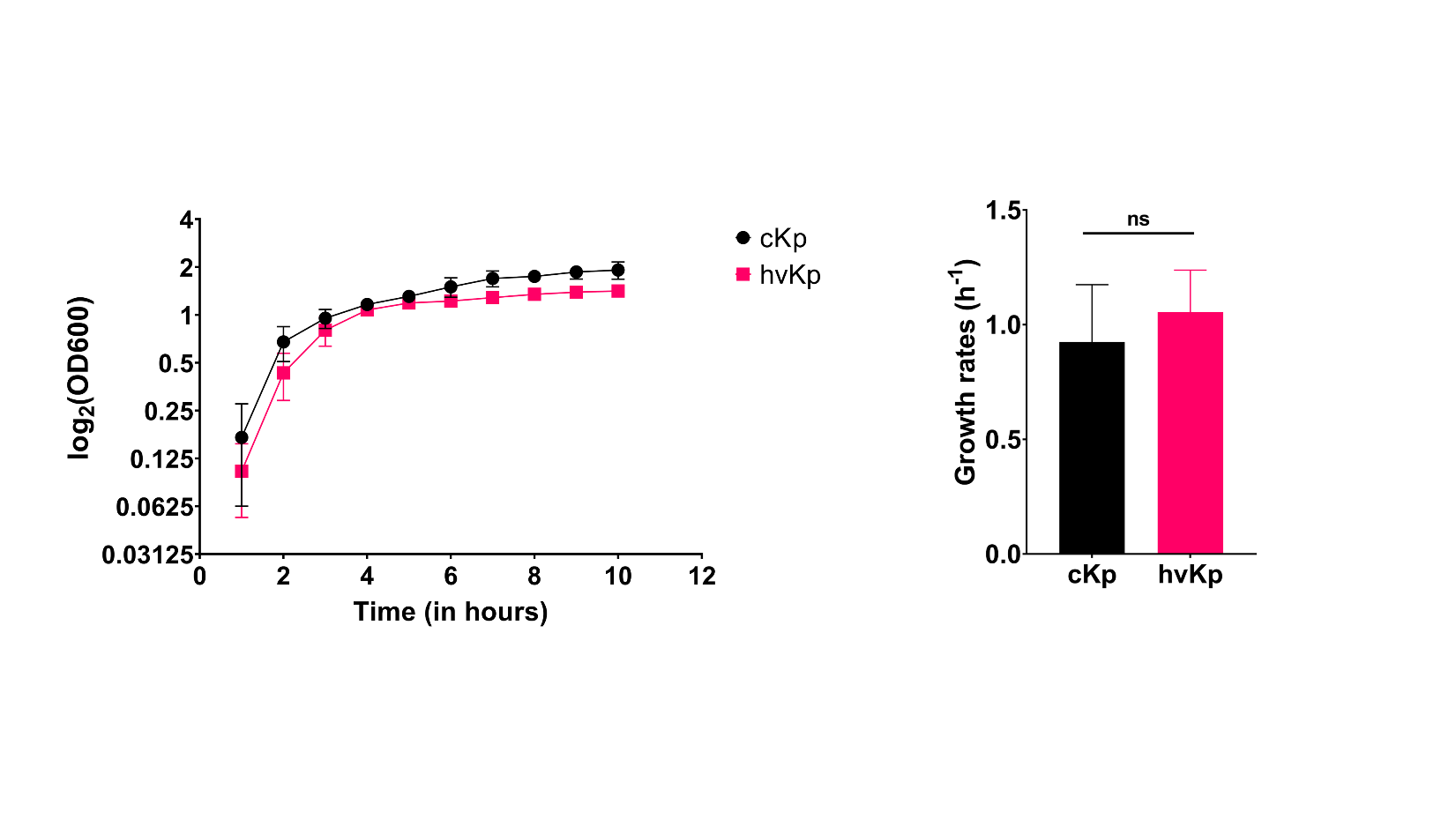


**A**

**B**

***Supplementary Figure 1.*** ***Growth kinetics of classical (cKp) and hypervirulent K.*** ***pneumoniae (hvKp). (A)*** *Growth curves of cKp (n=3: cKp01, cKp05, cKp06) and hvKp (n=6: hvkp01, hvKp02, hvKp03, hvKp04, hvKp07, hvKp09) isolates sequenced here. (B) Growth rates (hour^-1^) of cKp and hvKp isolates. No significant difference in growth rates were observed between the two pathotypes grown under laboratory conditions at 37^o^C in Luria Broth.*

***Supplementary Figure 2.*** ***Box plots depicting frequencies of GATC and CCWGG motifs.*** *GATC* ***(A)*** *and CCWGG* ***(B)*** *motif frequencies per Kb length found in contigs > 5Mb (chromosomes) and < 5 Mb (Putative extra-chromosomal genetic elements, PEGEs) for both classical K. pneumoniae (cKp; n=5) and hypervirulent K. pneumoniae (hvKp; n=6) isolates sequenced. * P<0.01.*


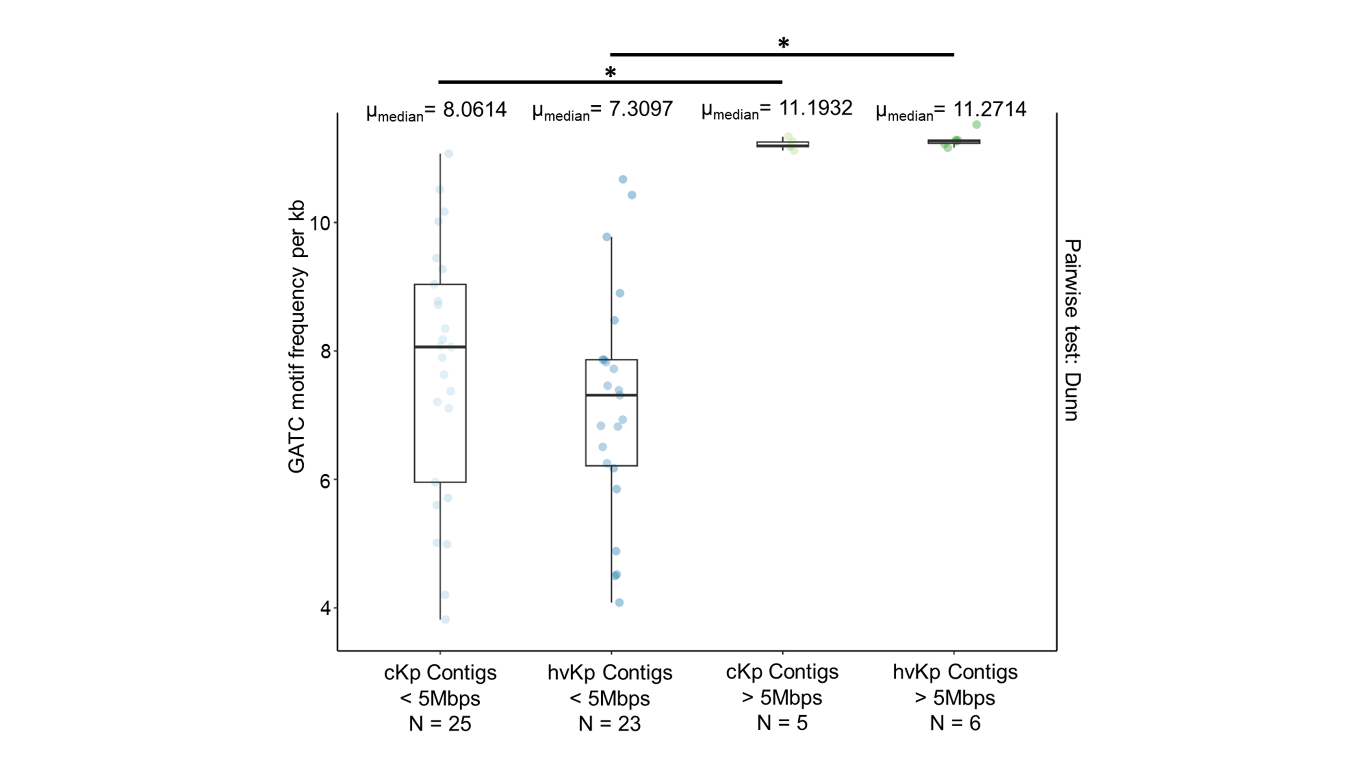

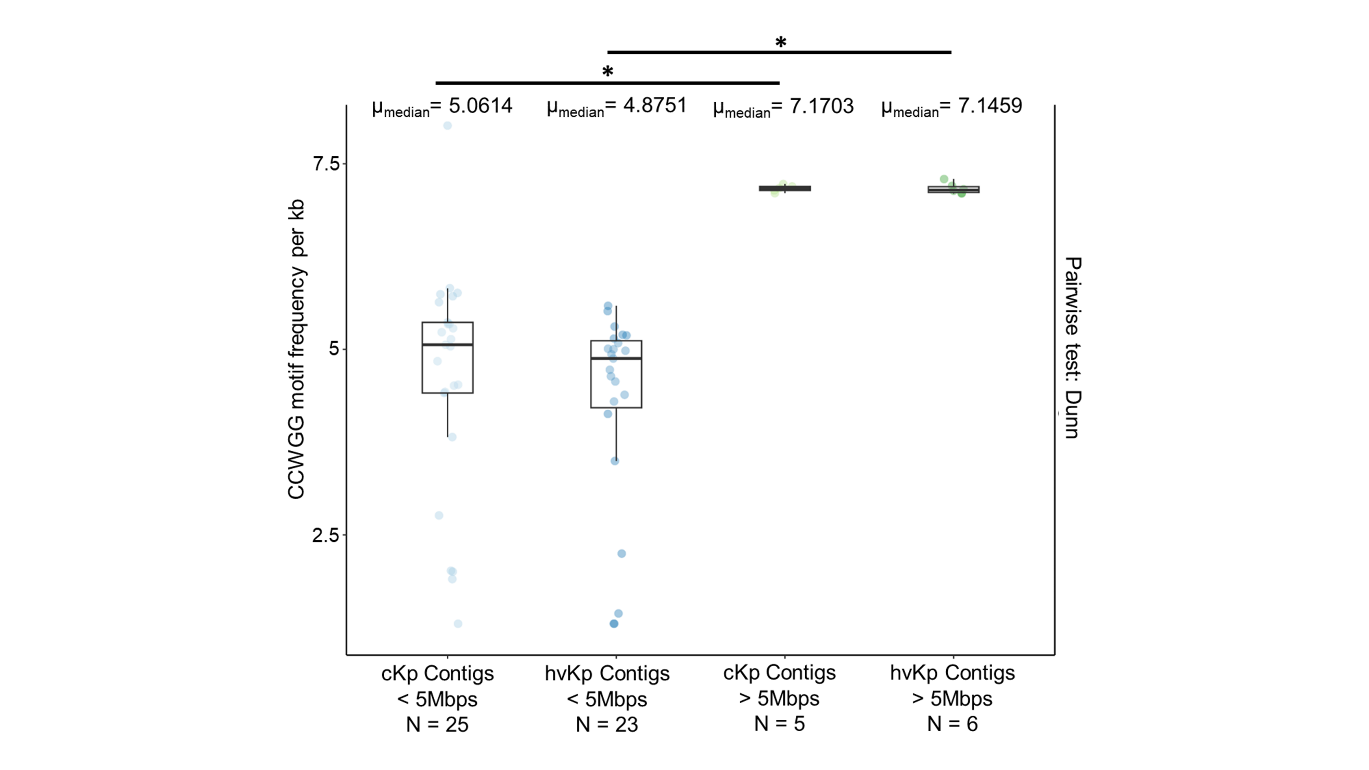


**A**

**B**


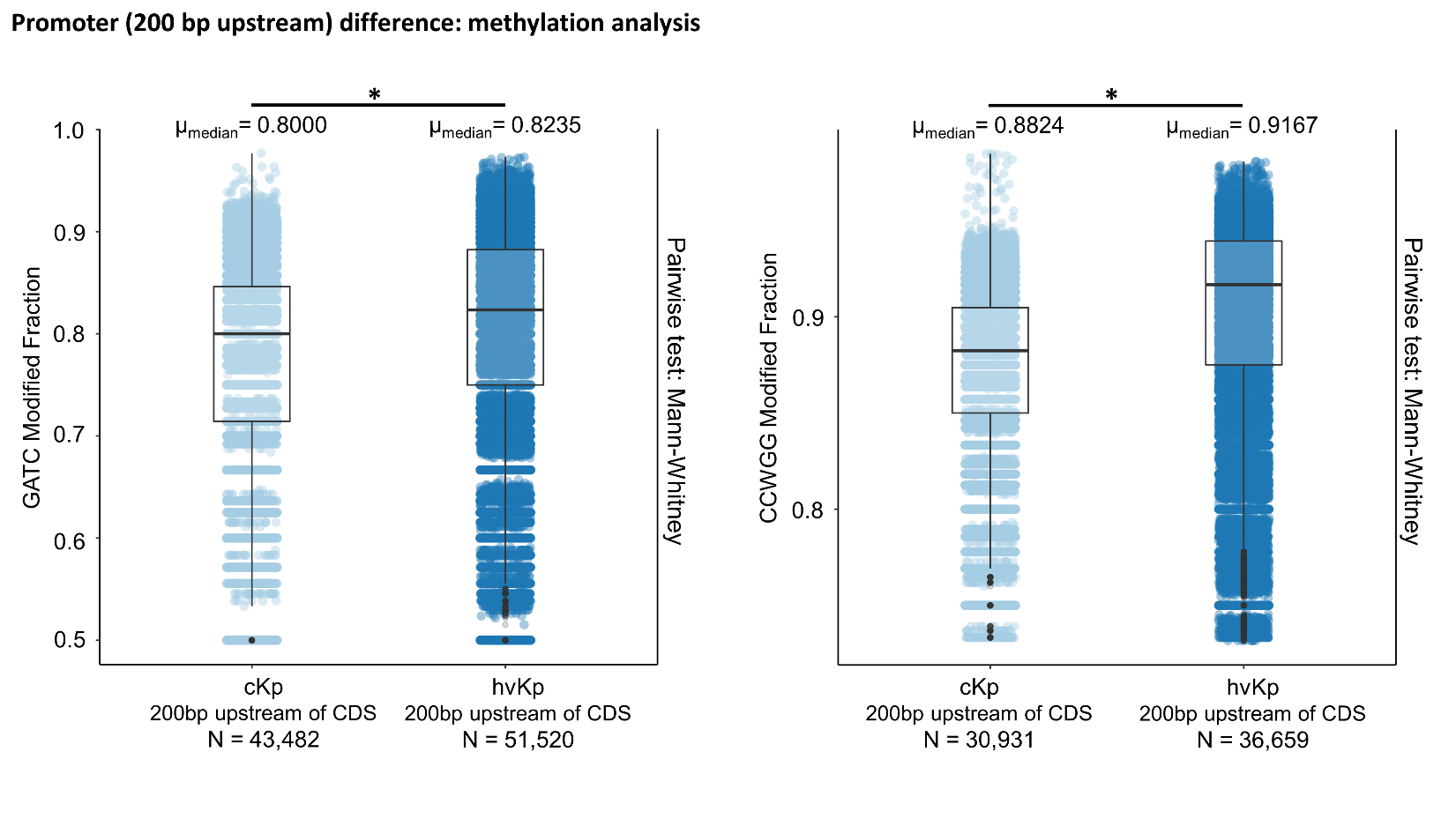
***Supplementary Figure 3.*** ***Box plots depicting methylation of GATC and CCWGG motifs upstream of gene coding sequences (CDS).*** *GATC* ***(A)*** *and CCWGG* ***(B)*** *modified fraction (ModFrac) values 200 bases upstream of CDS sequences in classical (cKp) and hypervirulent (hvKp) pathotypes of K. pneumoniae. (Outliers, defined using 1.5 times Interquartile Range method, have been removed). * P<0.0001.*

**B**

**A**


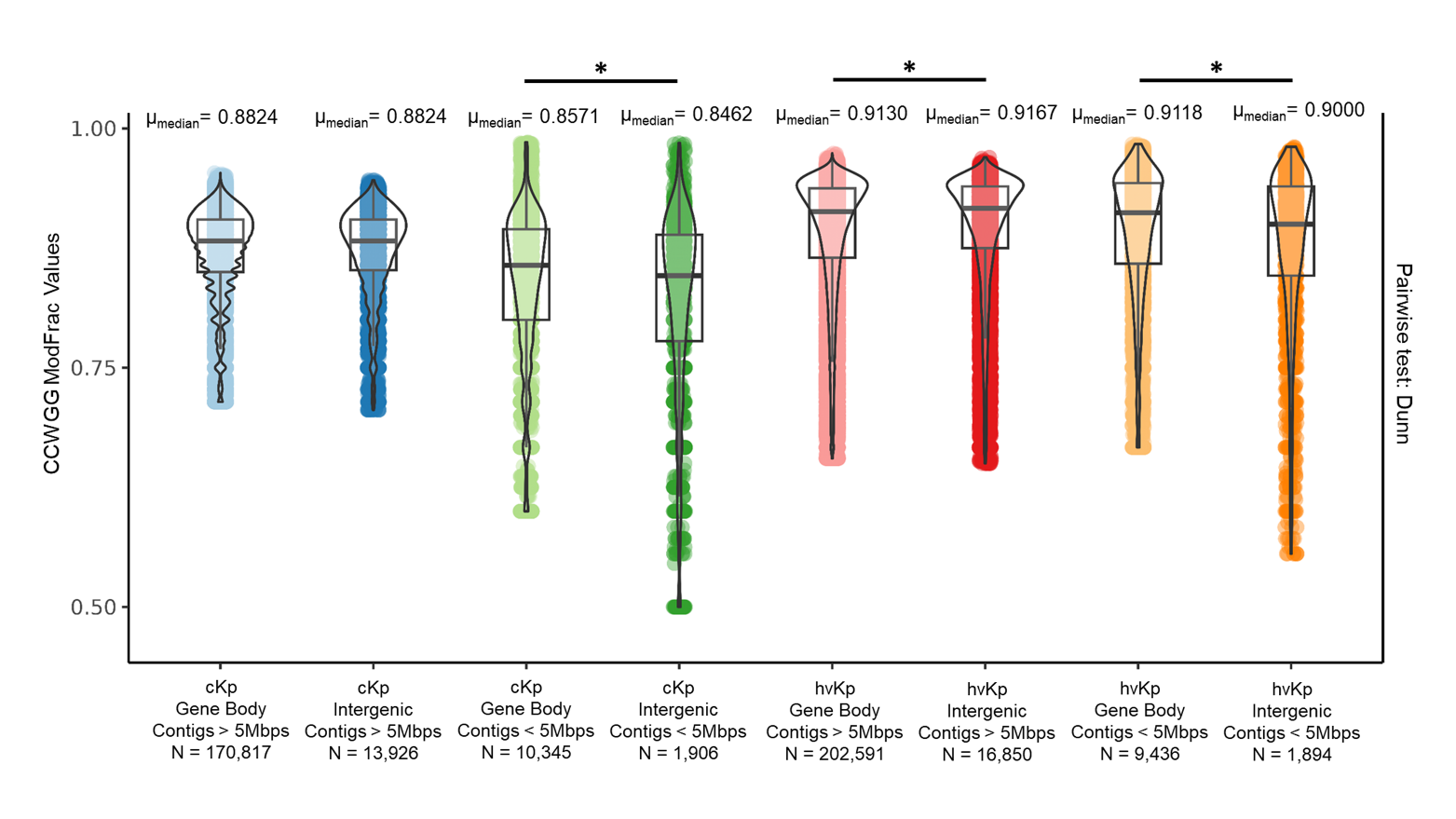

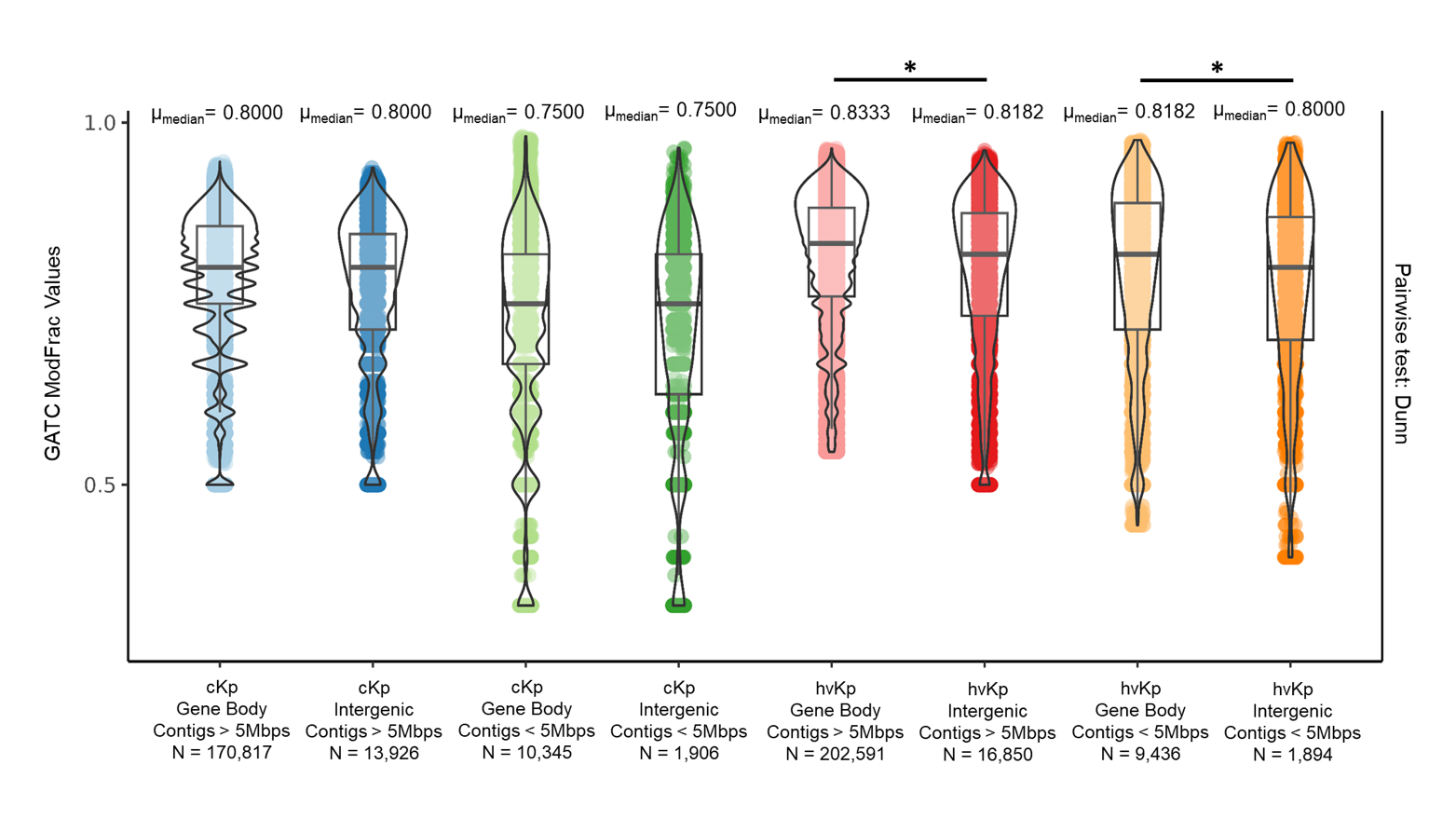
***Supplementary Figure 4.*** ***Comparison of methylation levels of GATC and CCWGG motifs found in gene bodies and intergenic regions of classical K. pneumoniae (cKp) and hypervirulent K. pneumoniae (hvKp) genomes.*** *Violin plots depicting distribution of GATC* ***(A)*** *and CCWGG* ***(B)*** *ModFrac values across gene bodies and intergenic regions in the assemblies of cKp and hvKp sequenced in this study. (Outliers, defined using 1.5 times Interquartile Range method, have been removed). * P<0.0001.*

**A**

**B**


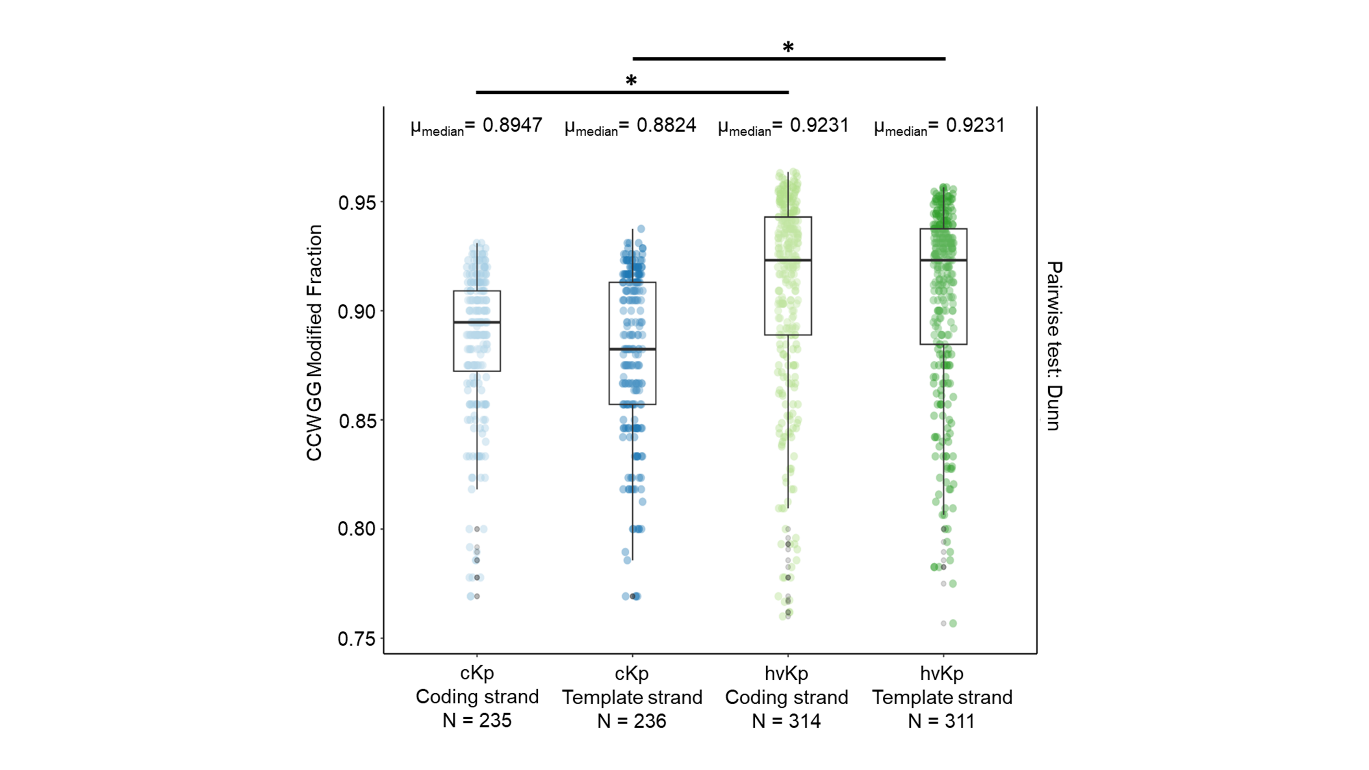

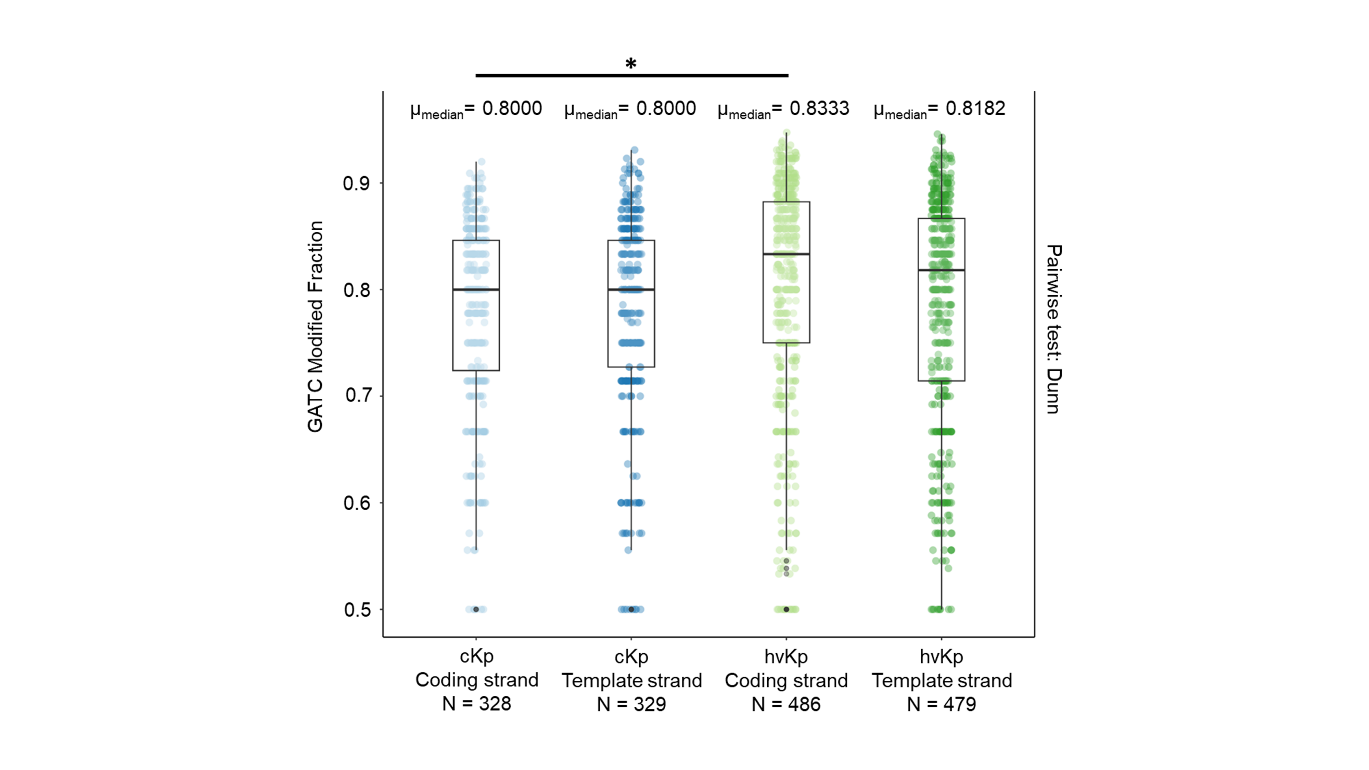
***Supplementary Figure 5.*** ***Comparison of methylation profiles of capsule synthesis locus in classical K. pneumoniae (cKp) and hypervirulent K. pneumoniae (hvKp).*** *Box plots summarizing the methylation profiles of GATC* ***(A)*** *and CCWGG* ***(B)*** *motifs present within the capsule synthesis locus (cps locus) of both cKp and hvKp. (Outliers, defined using 1.5 times Interquartile Range method, have been removed). * P<0.0001.*
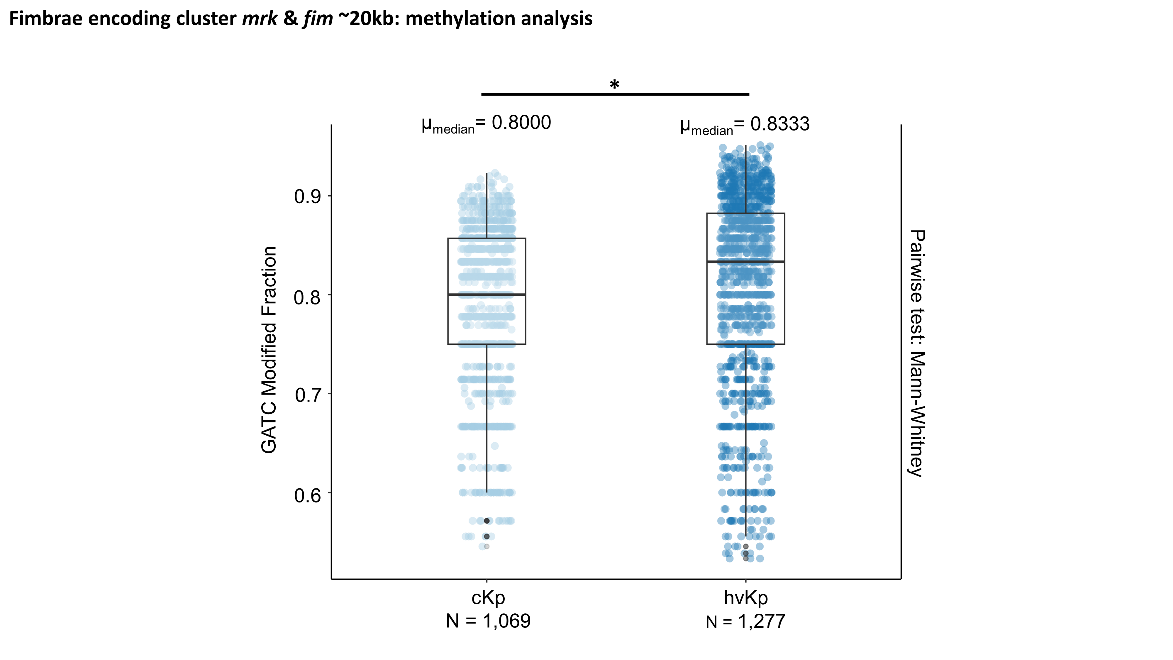

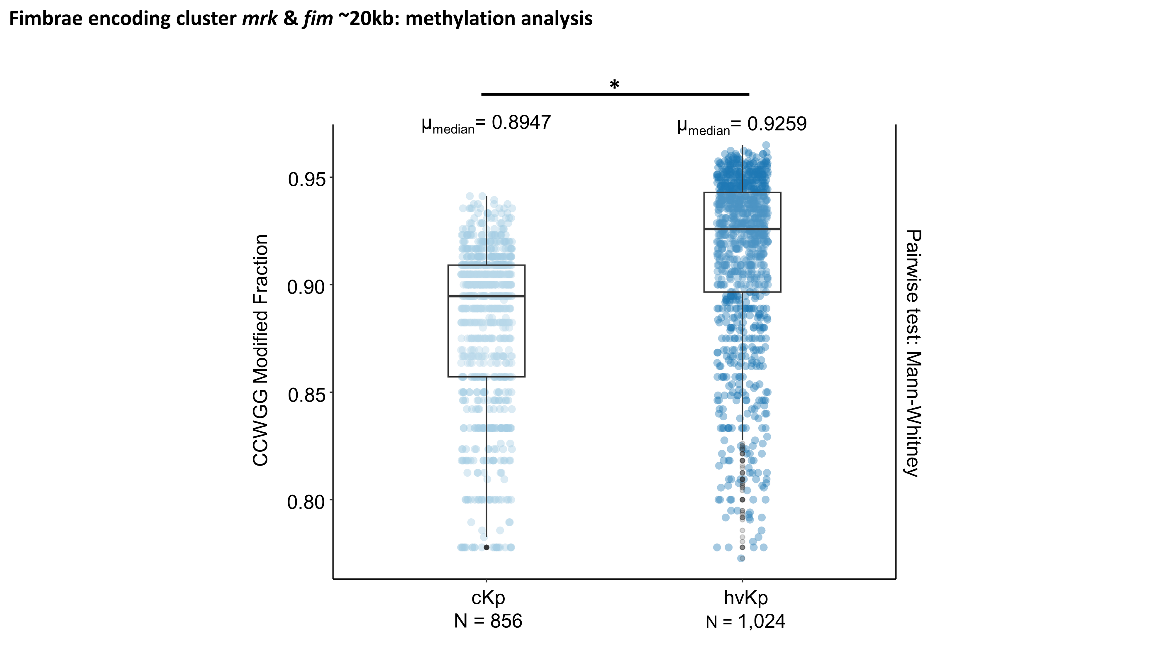
***Supplementary Figure 6.*** ***Comparison of methylation profiles of fimbriae-encoding cluster in classical K. pneumoniae (cKp) and hypervirulent K. pneumoniae (hvKp).*** *Box plots summarizing the methylation profiles of GATC* ***(A)*** *and CCWGG* ***(B)*** *motifs present within the conserved fimbriae-encoding cluster in cKp and hvKp. This cluster includes both Type 3 (mrk) genes as well as Type 1 (fim) genes. (Outliers, defined using 1.5 times Interquartile Range method, have been removed). * P<0.0001.*

**A**

**B**

**A**

**B**


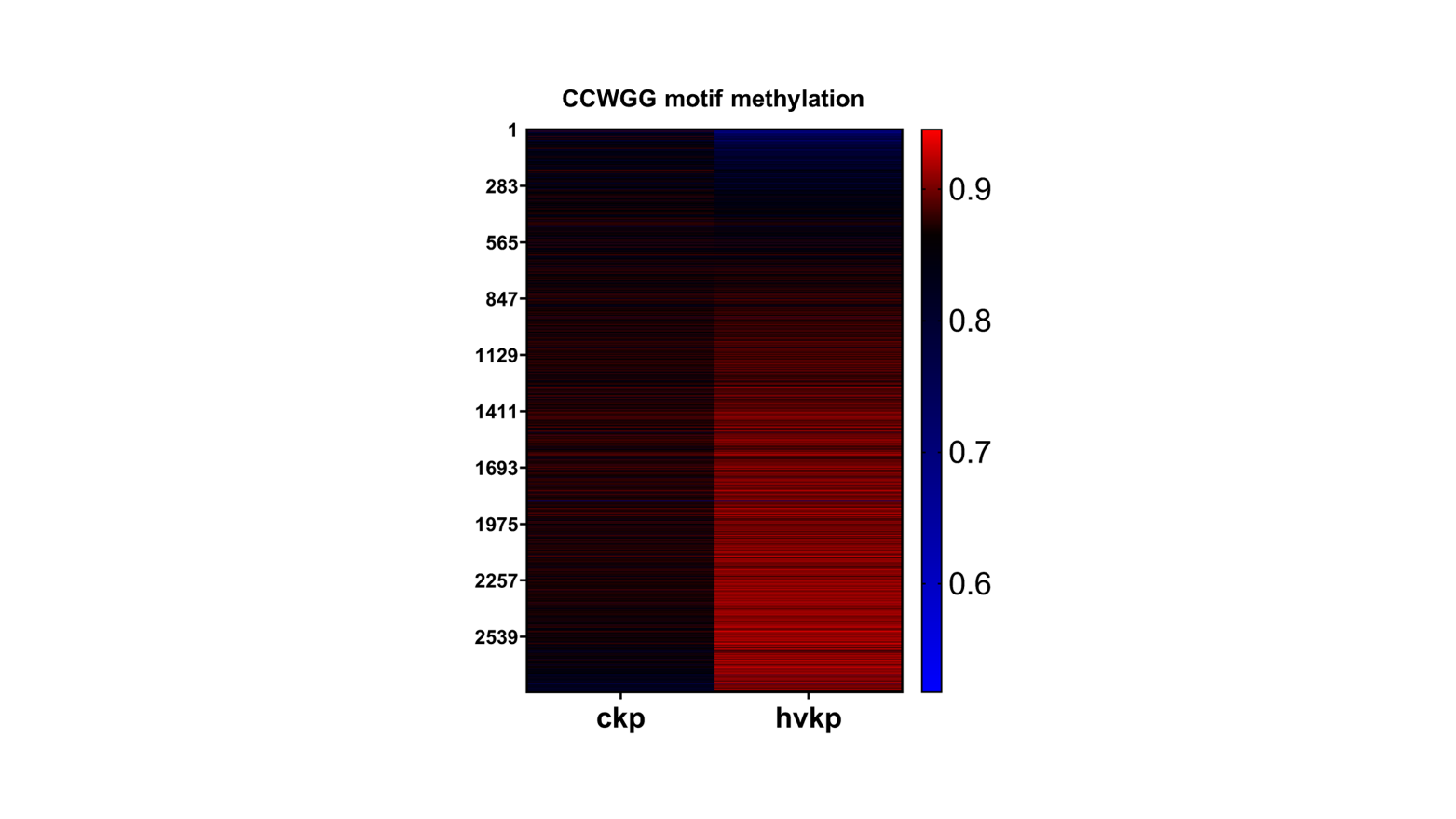

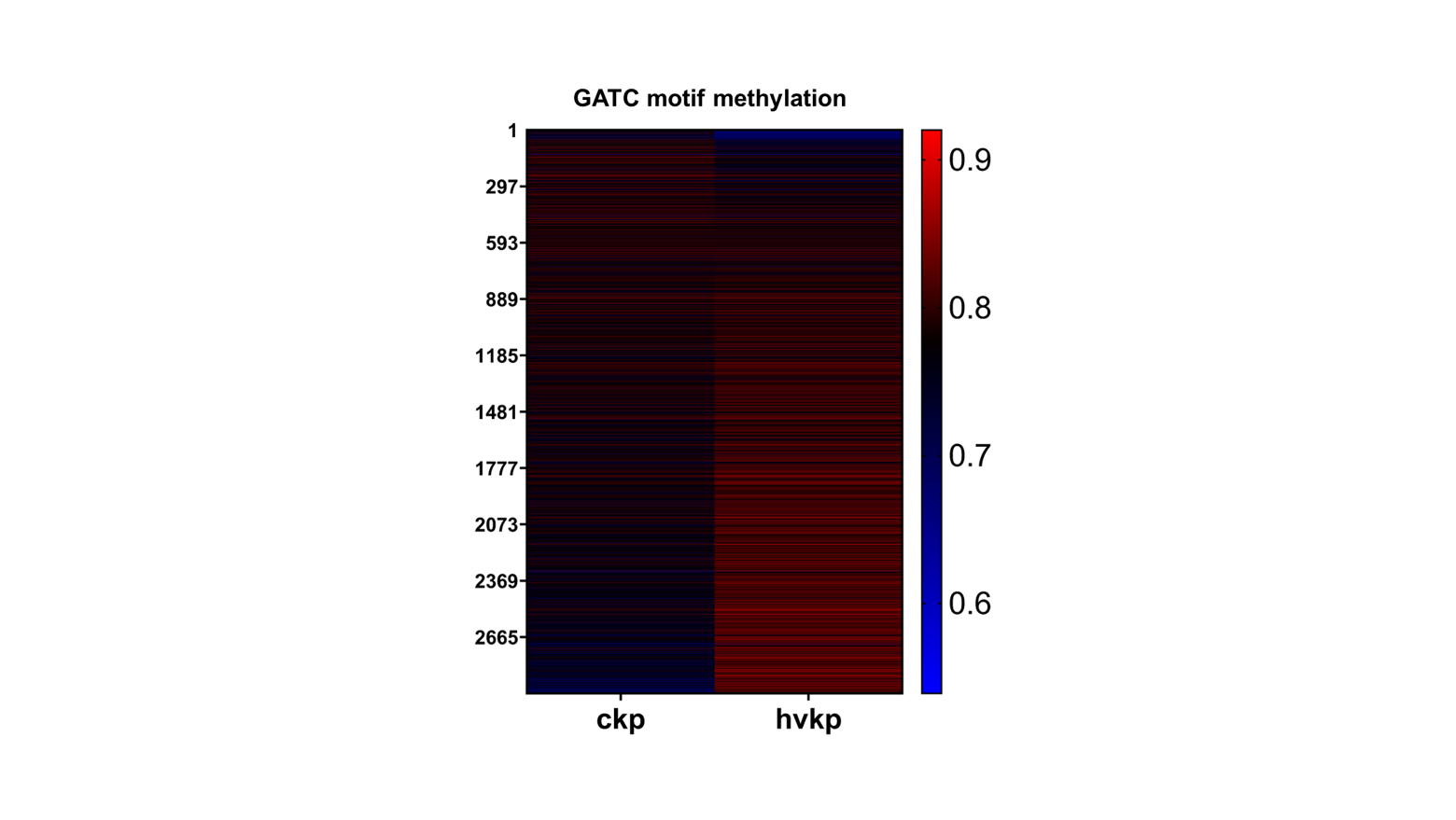
***Supplementary Figure 7.*** ***Differentially methylated genes in hypervirulent K. pneumoniae (hvKp). (A)*** *&* ***(B)*** *Three-color heatmaps depicting the average ModFrac values of GATC (A) and CCWGG (B) motifs present across each of the core genes of classical K. pneumoniae (cKp) and hvKp. Each row of the heatmaps correspond to a core gene and the color scales represent the averaged ModFrac value of a core gene (maximum=red, median=black, minimum=blue).* ***(C)*** *&* ***(D)*** *The z-scores of the difference in the methylation levels of core genes between cKp and hvKp have been plotted for both GATC (C) and CCWGG (D) motifs. Hypermethylated genes (z-score ≥2) are marked in red, while hypomethylated genes (z-score ≤ 2) are highlighted in blue.*

**A**

**B**


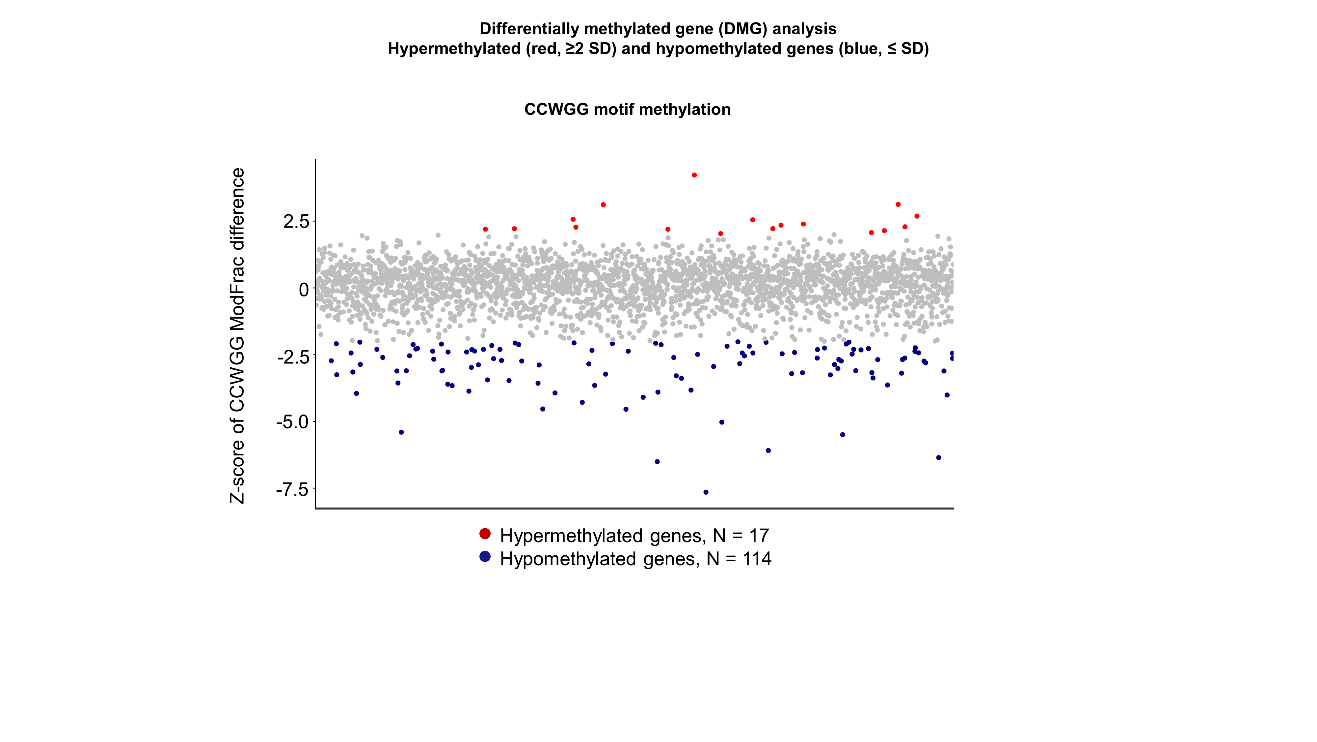

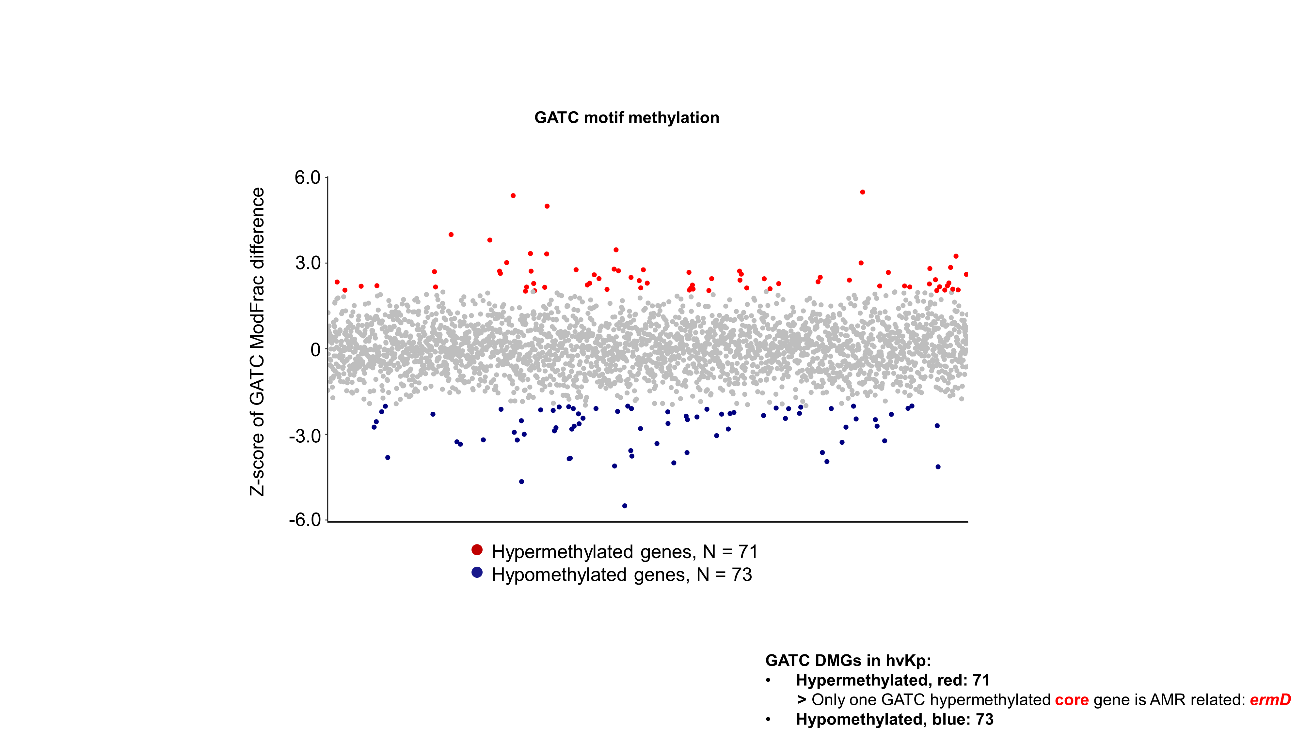


**D**

**C**

**SUPPLEMENTARY TABLES**


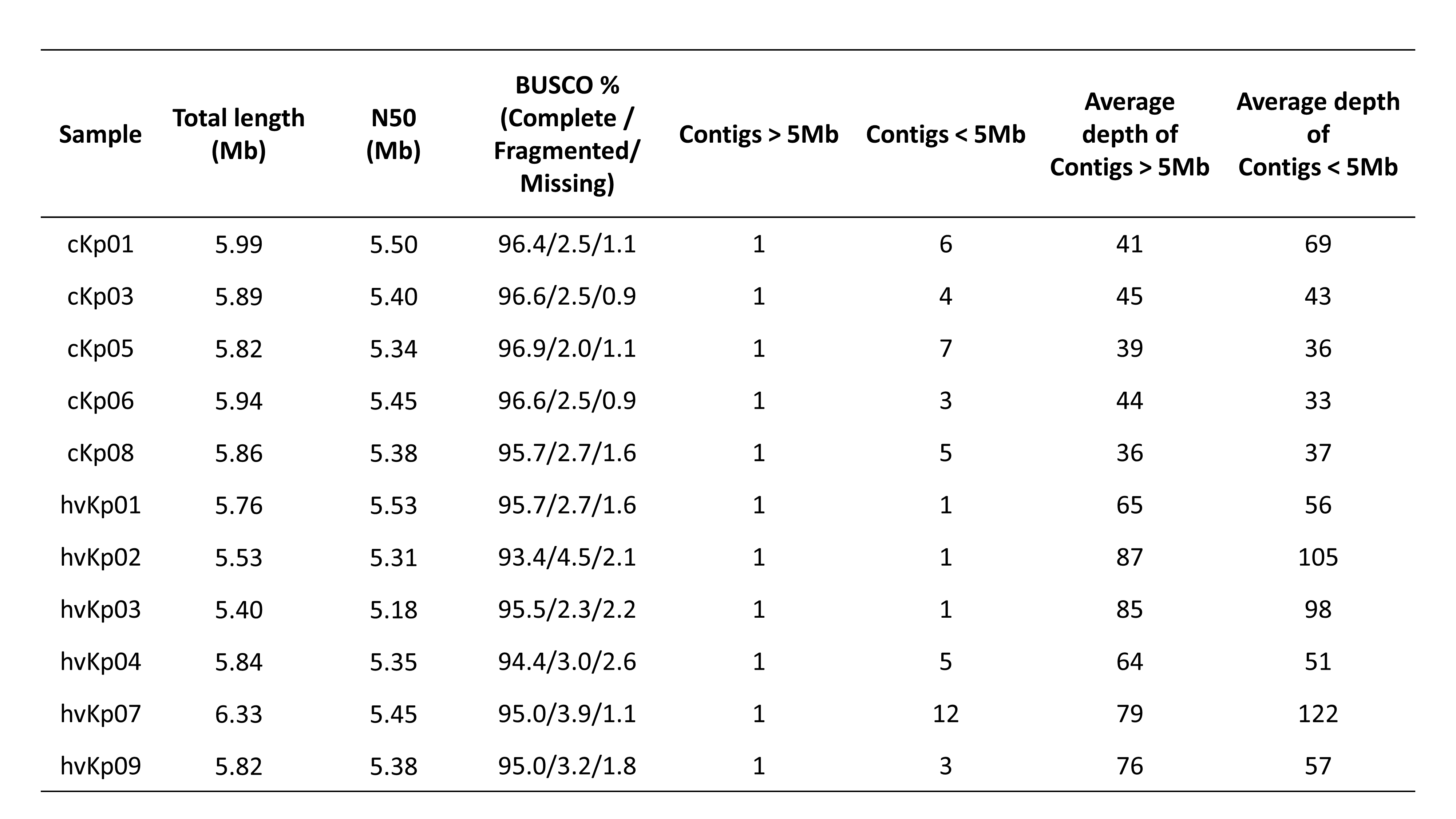
***Supplementary Table 1.*** *Statistics of de novo assemblies of classical (cKp) and hypervirulent (hvKp) Klebsiella pneumoniae isolates.*

***Supplementary Table 2.*** *Differentially methylated genes for GATC* ***(A)*** *and CCWGG* ***(B)*** *in hypervirulent Klebsiella pneumoniae (hvKp) involved in inorganic ion transport and metabolism.*

**A**

**B**


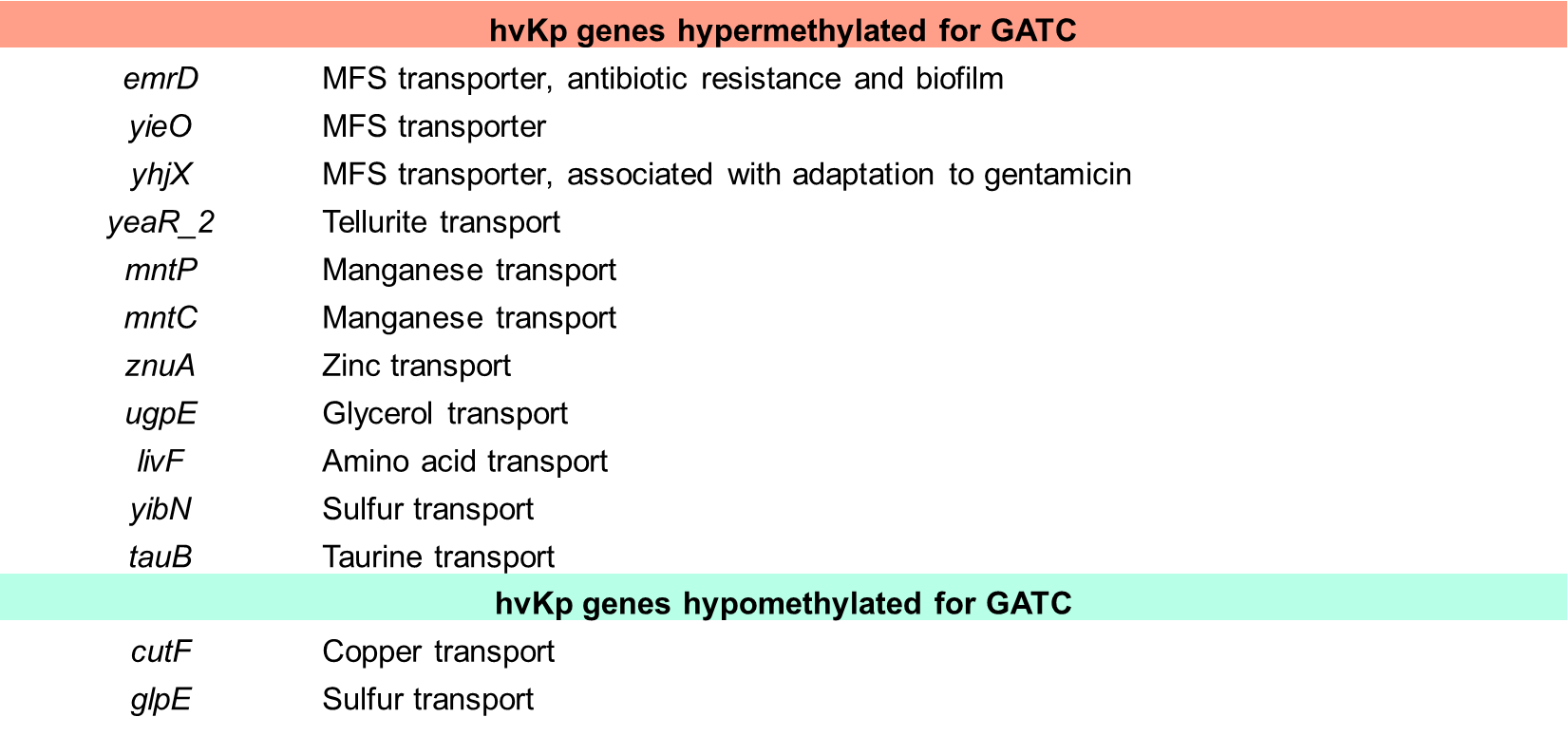

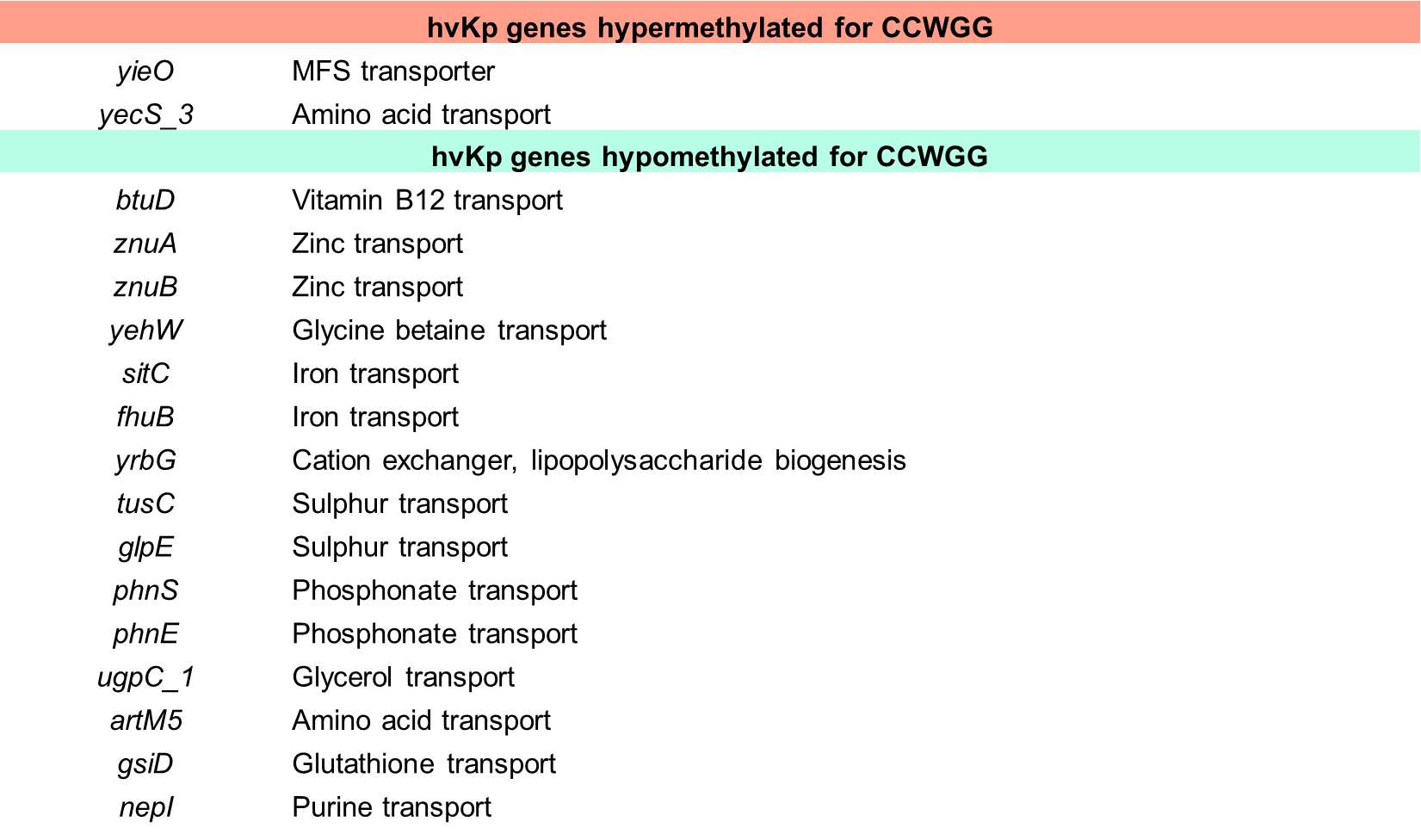


***Supplementary Table 3.*** *Differentially methylated genes for GATC* ***(A)*** *and CCWGG* ***(B)*** *in hypervirulent Klebsiella pneumoniae (hvKp) involved in transcription.*


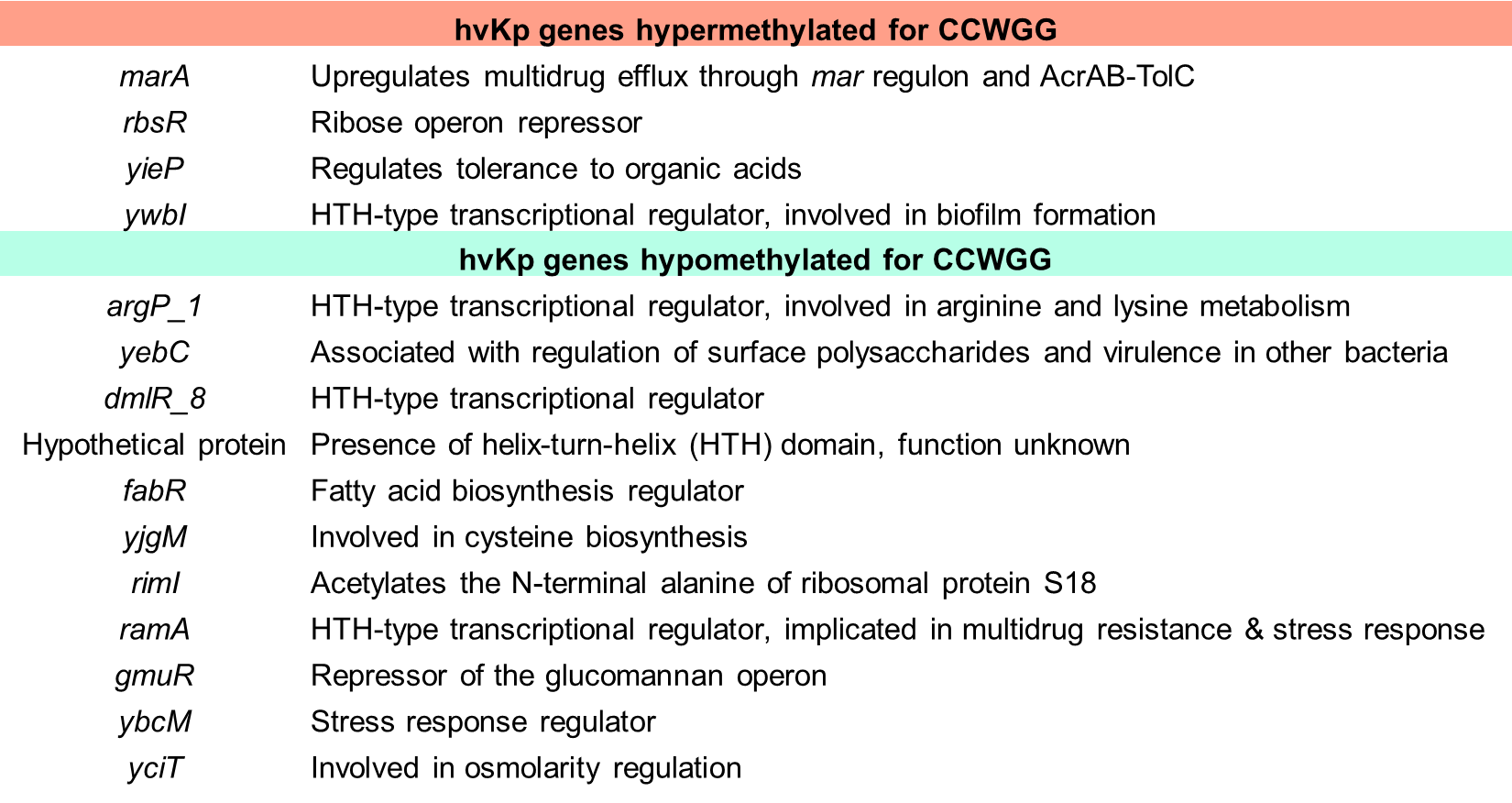

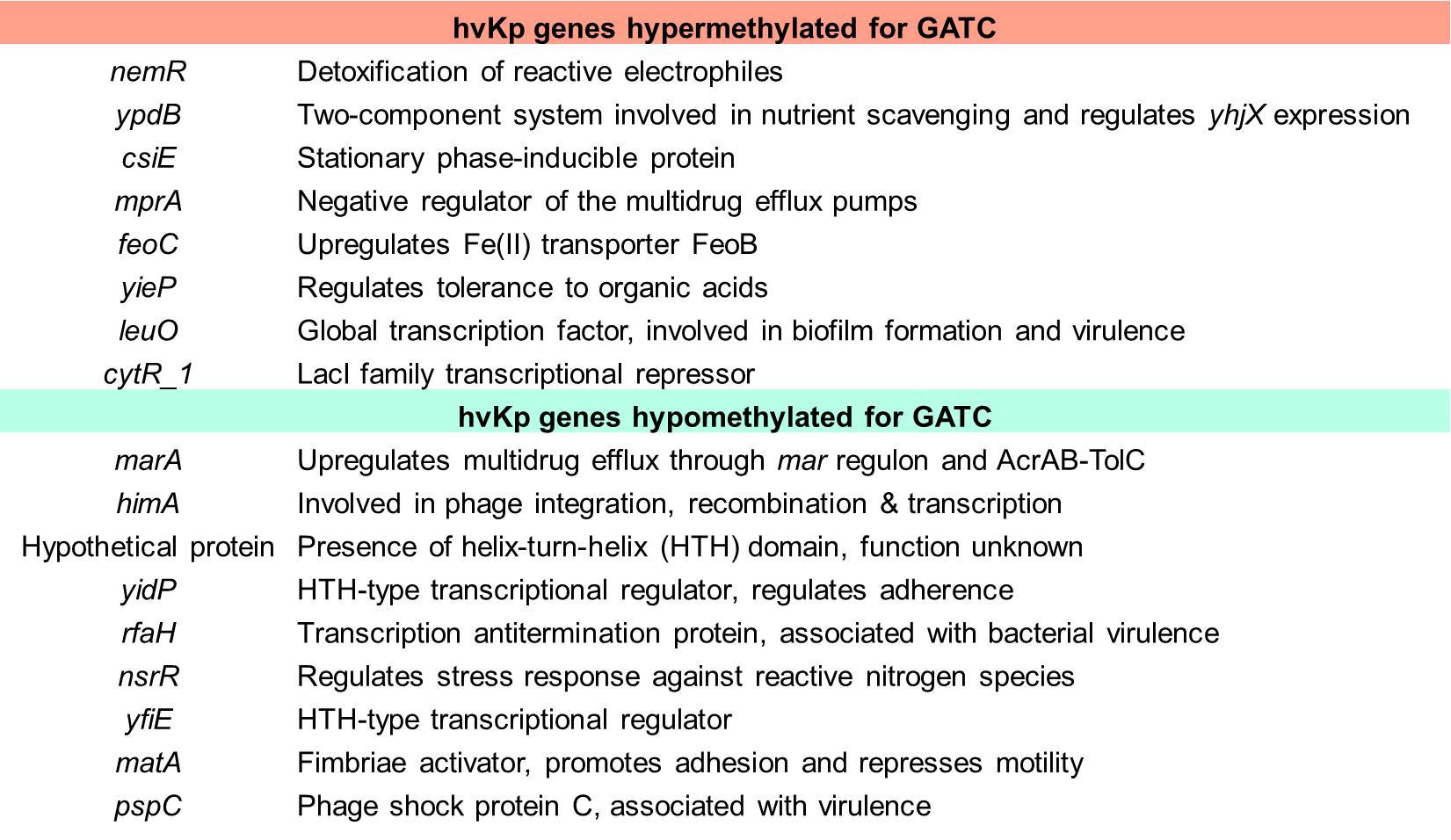


**A**

**B**
